# Equating scores of the University of Pennsylvania Smell Identification Test and Sniffin' Sticks test in patients with Parkinson's disease

**DOI:** 10.1016/j.parkreldis.2016.09.023

**Published:** 2016-12

**Authors:** Michael Lawton, Michele T.M. Hu, Fahd Baig, Claudio Ruffmann, Eilidh Barron, Diane M.A. Swallow, Naveed Malek, Katherine A. Grosset, Nin Bajaj, Roger A. Barker, Nigel Williams, David J. Burn, Thomas Foltynie, Huw R. Morris, Nicholas W. Wood, Margaret T. May, Donald G. Grosset, Yoav Ben-Shlomo

**Affiliations:** a School of Social and Community Medicine, University of Bristol, United Kingdom; b Nuffield Department of Clinical Neurosciences, Division of Clinical Neurology, University of Oxford, United Kingdom; c Oxford Parkinson's Disease Centre, University of Oxford, United Kingdom; d Department of Neurology, Institute of Neurological Sciences, Queen Elizabeth University Hospital, Glasgow, United Kingdom; e Department of Neurology, Queen's Medical Centre, Nottingham, United Kingdom; f Clinical Neurosciences, John van Geest Centre for Brain Repair, Cambridge, United Kingdom; g Institute of Psychological Medicine and Clinical Neurosciences, Cardiff University, United Kingdom; h Institute of Neuroscience, University of Newcastle, United Kingdom; i Sobell Department of Motor Neuroscience, UCL Institute of Neurology, United Kingdom; j Department of Clinical Neuroscience, UCL Institute of Neurology, United Kingdom; k Department of Molecular Neuroscience, UCL Institute of Neurology, United Kingdom

**Keywords:** Olfaction, Sniffin’ Sticks, University of Pennsylvania Smell Identification Test, Equating, Item Response Theory

## Abstract

**Background:**

Impaired olfaction is an important feature in Parkinson's disease (PD) and other neurological diseases. A variety of smell identification tests exist such as “Sniffin’ Sticks” and the University of Pennsylvania Smell Identification Test (UPSIT). An important part of research is being able to replicate findings or combining studies in a meta-analysis. This is difficult if olfaction has been measured using different metrics. We present conversion methods between the: UPSIT, Sniffin’ 16, and Brief-SIT (B-SIT); and Sniffin’ 12 and Sniffin’ 16 odour identification tests.

**Methods:**

We used two incident cohorts of patients with PD who were tested with either the Sniffin’ 16 (n = 1131) or UPSIT (n = 980) and a validation dataset of 128 individuals who took both tests. We used the equipercentile and Item Response Theory (IRT) methods to equate the olfaction scales.

**Results:**

The equipercentile conversion suggested some bias between UPSIT and Sniffin’ 16 tests across the two groups. The IRT method shows very good characteristics between the true and converted Sniffin’ 16 (delta mean = 0.14, median = 0) based on UPSIT. The equipercentile conversion between the Sniffin’ 12 and 16 item worked well (delta mean = 0.01, median = 0). The UPSIT to B-SIT conversion showed evidence of bias but amongst PD cases worked well (mean delta = −0.08, median = 0).

**Conclusion:**

We have demonstrated that one can convert UPSIT to B-SIT or Sniffin’ 16, and Sniffin’ 12 to 16 scores in a valid way. This can facilitate direct comparison between tests aiding future collaborative analyses and evidence synthesis.

## Highlights

• Need to harmonise olfaction tests (UPSIT and Sniffin’ Sticks) for meta-analysis.
• Use of Item Response Theory and equipercentile methods to convert scores.
• Validation shows high concordance and little bias between true and converted scores.
• New age and gender corrected centiles for impaired olfaction from Sniffin’ scores.

## 1 Introduction

Impaired olfaction is an important non-motor feature of Parkinson's disease (PD). It is thought to be an early pre-clinical sign of PD [1] and can be used to help in the diagnosis of PD before the development of definite motor features [2], [3]. Olfactory impairment may also be an early marker of other neurological diseases such as Alzheimer's disease [4], multiple sclerosis [5], idiopathic rapid eye movement sleep behaviour disorder [6], Huntington's disease [7], multiple system atrophy [8], progressive supranuclear palsy [9] and parkinsonism dementia complex seen in Guam [10]. Differences in olfactory dysfunction between neurological diseases may be helpful in the differential diagnosis [11] of parkinsonian disorders [12]. Detailed reviews of olfactory dysfunction in neurological disorders have been previously published [11], [13].

Many research studies collect data on olfaction and an important aspect of high quality research is the ability to replicate findings from studies or undertaking systematic reviews with or without a meta-analysis to synthesise evidence and examine for heterogeneity. This is more difficult if olfaction has been measured using a different metric within the different studies leading to potentially artefactual differences. The ability to estimate scores on one test from scores on another test helps reduce this problem. Olfaction is often measured using smell identification tests such as Sniffin’ Sticks [14] or the University of Pennsylvania Smell Identification Test (UPSIT) [15].

Both the Sniffin’ [16] and UPSIT [17] tests have published normative data centiles stratified by age and gender allowing us to determine the olfactory changes that are likely to be caused by disease in addition to that due to the natural aging process. This is particularly important in PD which predominantly affect the older population. Whilst the published normative data for Sniffin’ stratified age as 5–15; 16–35; 36–55; and >55, the UPSIT stratified using five year age bands up to 85 and above. The stratification method employed by UPSIT is arguably more sensible given that olfactory impairment rises dramatically between 65 and 80 years [18].

We aimed to create conversion tables from an UPSIT score to a standard Sniffin’ 16 item odour identification score, between the Sniffin’ 12 and 16 item odour identification versions and between the UPSIT and Brief Smell Identification test (B-SIT) using two large cohorts of individuals with PD to help researchers pool data in future collaborative studies. An additional useful by-product of our conversion is that we can convert the published age/gender stratified centiles for the UPSIT to equivalent Sniffin’ scores.

## 2 Methods

### 2.1 Study populations

Data were available from two incidence cohorts of patients with PD. The Oxford Parkinson's Disease Centre Discovery cohort consists of individuals from 11 hospitals across the Thames Valley. Patients were recruited between study onset in September 2010 up to May 2015. Full details of this study are described in detail elsewhere [19]. Patients were eligible for study inclusion if they met the UK PD Brain Bank Criteria according to a neurologist with a special interest in PD. We included any individuals diagnosed within the last three and a half years and who were given a probability of PD ≥ 90% as rated by a clinician based on their clinical opinion. This was to try to eliminate the inclusion of similar conditions that have been incorrectly diagnosed as PD. All individuals in this study had their olfaction measured using the standard Sniffin’ test.

Tracking Parkinson's is a large incidence cohort of patients with PD recruited from around the UK. Patients were recruited between February 2012 and May 2014 if they were diagnosed within the last 3.5 years and met Queen Square Brain Bank criteria. Full details of this study are described elsewhere [20]. Again we only included individuals who were given a probability of PD ≥ 90% as rated by a clinician. In this cohort, olfaction was initially measured using the UPSIT. However during the course of the study a difficulty arose in obtaining the UPSIT kits and the study was forced to switch to using the Sniffin’ test instead. This means we have two groups of individuals within the same cohort completing different tests.

We also have a third dataset of subjects “Testing of olfaction in Parkinson's and controls” (TOPC) who undertook both tests (Sniffin’ and UPSIT) concurrently so we could validate our conversion algorithms. This comprised of 128 subjects (61 PD and 67 controls) who were recruited as a convenience sample from the regional, West of Scotland, Movement Disorder Clinic. The order on which individuals took the two tests was randomised thus minimising any order effects, such as patients scoring worse on the second test due to fatigue.

All three studies had ethical approval and were undertaken with the understanding and written consent of each subject and in compliance with the declaration of Helsinki.

### 2.2 Olfaction tests

The UPSIT test has 40 items, where each item has one correct answer and three incorrect answers or “distractors”. The test is a forced choice paradigm, that is, if an individual is unsure of an answer they are forced to guess a response hence a score of 25% on average would reflect random guessing. An UPSIT result is scored out of 40 where a higher score indicates better olfaction. There is also a reduced 12 item version [21] of the UPSIT called the Brief-Smell Identification Test (B-SIT), previously called the Cross-cultural Smell Identification Test (CC-SIT).

The standard Sniffin’ test has 16 odour identification items, where each item has one correct answer and three incorrect answers or “distractors”. Again the test is a forced choice paradigm. A Sniffin’ result is scored out of 16 where a higher score indicates better olfaction. There is also a Sniffin’ 12 item version [22] which is a subset of the 16 item version.

### 2.3 Statistical analysis

The first and simplest method of equating one scale to another is equipercentile equating with log-linear smoothing which matches scores on the two tests using their percentile ranks after first smoothing the distribution. This method requires that the two groups are equivalent in olfaction usually through design creating randomly equivalent groups or by carrying out both tests on the same population. In our case it would mean assuming the groups taking the Sniffin’ and UPSIT tests are equivalent with regards to olfaction.

Our second method used Item Response Theory (IRT) which models individual's responses on the item level by fitting a series of latent variable models for each item. The power of the IRT approach is that we calibrated our model between groups with potentially different olfaction by using items that are common to both tests. We assumed that the two groups are linearly related by their olfaction and calculated a calibration slope and intercept between the two groups. After calibration we built the distribution of scores and then equated using equipercentile methods.

Both the equipercentile and IRT methods are described in detail by Kolen and Brennan [23] whilst the details of how we used the IRT method and the computing programs we used are discussed further in the Web appendix.

We used both methods to convert between the UPSIT and Sniffin’ 16 item test. Since the Sniffin’ 12 items is a subset of the Sniffin’ 16 item and the B-SIT is a subset of the UPSIT they were carried out on the same population. Hence we only used the equipercentile method for the UPSIT to B-SIT and Sniffin’ 12 to 16 item conversions. We used our validation dataset to test how well the conversions performed by comparing the concordance correlation coefficient [24] (a measure of agreement between two continuous variables) between true and equivalent results as well as the characteristics of the difference (or delta) between the true and equivalent.

We also converted the centile position stratified by age and gender from the UPSIT normative data charts to an equivalent Sniffin’ score to provide more detailed normative comparative data. We used at or below the 15th centile as a cut-point for determining whether an individual has impaired olfaction corrected for age and gender as we have done in previous research [25]. There are some inconsistent and random fluctuations in the centiles (probably due to sample size issues) hence we used LOWESS techniques to smooth the cut-points before applying our conversion.

## 3 Results

### 3.1 Demographic and clinical data for Tracking Parkinson's and Oxford Discovery cohorts

Table 1 compares the data we have from the Tracking Parkinson's with 980 individuals who took the UPSIT test and 294 who took the Sniffin’ test at the baseline visit. These two sub-groups of the Tracking Parkinson's cohort have a similar proportion of females, age when the testing took place, motor severity (measured by the Movement Disorder Society Unified PD Rating Scale or MDS-UPDRS part 3), disease severity (measured by Hoehn and Yahr stage) and cognitive impairment (measured by the education adjusted Montreal Cognitive Assessment or MoCA). However the UPSIT sub-group had slightly longer disease duration. This is not surprising given that the UPSIT sub-group would have been recruited first in the study, which would include both incident and some prevalent cases (up to 3.5 years), however the cases that are recruited later on in the centres would consist of mainly incident cases since the prevalent pool of cases would have already been recruited.

In the Oxford Discovery cohort we have 837 individuals who took the Sniffin’ 16-item odour identification test at the baseline visit. When compared to the group who took the UPSIT test from the Tracking Parkinson's cohort they had slightly shorter disease duration, a similar proportion of females and similar age at testing. They also had worse motor severity, disease severity and more cognitive impairment. Comparing the Tracking Parkinson's Sniffin’ subset and Oxford Discovery groups they show similar gender, age and cognitive impairment but Oxford Discovery has worse motor and disease severity and longer disease duration from diagnosis. Of paramount importance is that there is no evidence (p = 0.12) of a difference in Sniffin’ scores between the Tracking Parkinson's subset and Oxford Discovery groups. We therefore pooled the Sniffin’ data from the two cohorts for our UPSIT to Sniffin’ 16 conversion. Web table 1 shows the demographic data from the TOPC validation study and Web Fig. 2 shows the distribution of UPSIT and Sniffin’ 16 scores stratified by patient type. The correlation between the UPSIT and Sniffin’ 16 scores was 0.81 in this sample.

### 3.2 UPSIT to Sniffin’ 16 conversion

Table 2 shows the conversions from the UPSIT to a Sniffin’ 16 equivalent using the two methods. In general, most UPSIT scores were grouped into 2 point values equivalent to 1 Sniffin’ point but this could be as wide as 5 points for the (0–4) group using the IRT method. Table 3 presents the characteristics of these different conversions when tested on the TOPC validation data in which we compared an UPSIT predicted Sniffin’ 16 to a true Sniffin’ 16 score. The concordance correlation coefficient between the true and equivalent Sniffin’ is very good and similar using both the equipercentile (0.79) and IRT methods (0.80). The difference between equipercentile predicted and true Sniffin’ was acceptable although there was some evidence of under-prediction bias (positive mean delta). The individual IRT parameter estimates (*a, b, c*) for the UPSIT data and the combined Sniffin’ data can be found in Web Tables 2 and 3 When using the IRT method we found that the calibration slope was 1.093 and the calibration intercept was 0.180. This is equivalent to saying that the individuals taking the UPSIT test had marginally better olfaction and also a slightly larger spread of olfaction when compared to the Sniffin’ group. However mean olfaction that is 0.180 higher is small considering the groups are scaled to a mean of 0 and sd of 1. The validation of the IRT method on the TOPC data resulted in a delta that has a mean very close to zero and a median of zero showing that this conversion appears to have little evidence of bias. Web Fig. 3 shows graphically the degree of agreement between the true Sniffin’ and the UPSIT equivalent Sniffin’ using the two methods.

Comparison of these calibration estimates to the conversions carried out using the equipercentile method showed some agreement. Assuming these calibration estimates are correct implies that the olfaction was slightly different in the two populations and hence the assumptions for the equipercentile method do not hold. Considering these calibration estimates, individuals taking the UPSIT test seem to have slightly better olfaction when compared to the Sniffin’. In agreement with this the equipercentile method showed evidence of the difference in olfaction in the observed bias.

Table 4 shows the cut-points corresponding to the 15th centile of olfaction score stratified by age and gender from the UPSIT normative data. The table also shows the smoothed cut-points using LOWESS techniques and the equivalent Sniffin’ score when applying our conversion chart from the IRT method in Table 2. This allows researchers to define a binary hyposmic group (Yes/No) based on poor olfaction (≤15th centile) for each gender and different age groups which can be used in analyses testing predictors of hyposmia.

### 3.3 Sniffin’ 12 to Sniffin’ 16 conversion

In the conversion from Sniffin’ 12 to 16 we are no longer bound by assuming the groups to be equal because they are identical. This means that we can use data from each visit in the Discovery cohort rather than only using the baseline data. The number of individuals eligible for analysis were 837, 564, and 275 from visits 1, 2, and 3 respectively from the Discovery cohort along with the 294 from the Tracking Parkinson's cohort. The 1970 observations of combined Sniffin’ 16 data has a mean of 7.0 and s.d. of 2.8 whilst the combined Sniffin’ 12 data has a mean of 5.6 and s.d. of 2.4. Web table 4 shows the conversion scores from Sniffin’ 12 to a Sniffin’ 16 equivalent and Table 3 shows the validation of this conversion using the TOPC data. With these two tests being so similar it is not surprising that the concordance between true and equivalent Sniffin’ 16 was very high, 0.97, that the average delta between the two was so close to zero and the standard deviation of the delta was also low at 0.96. Web Fig. 4 shows graphically the degree of agreement using the true Sniffin’ 16 and the Sniffin’ 12 equivalent Sniffin’ 16. It could be argued that the percentiles used in the equipercentile method should not include an individual more than once, re-running this method using only the baseline data from the Discovery cohort gave an identical conversion.

### 3.4 UPSIT to B-SIT conversion

Web table 5 shows the conversion scores from UPSIT to B-SIT and Table 3 shows the validation of this conversion. The concordance coefficient is relatively high, 0.82, however when looking at the delta there is some evidence of over-prediction bias (negative average delta) in our conversion, mean = −0.63 and median = −1. However if we stratify the delta by PD cases (mean delta = −0.08 and median = 0) and controls (mean delta = −1.13 and median = −1) there is only evidence of bias for the controls. Web Fig. 5 shows graphically the degree of agreement using the true B-SIT and the UPSIT equivalent B-SIT.

## 4 Discussion

We used two methods to equate scores on the UPSIT test to scores on the Sniffin’ 16 smell identification test, scores on the Sniffin’ 12 item to Sniffin’ 16 item smell identification tests and also scores on the UPSIT and B-SIT tests.

It has been shown that the differences in olfaction between PD patients and controls is not related to any particular odour type [26]. This suggests that although our conversions have been created using only PD patients they could potentially be used for controls and/or other diseases where olfactory dysfunction is not related to particular odour types.

A previous paper reported that the correlation between the Sniffin’ and UPSIT scores was 0.85 [14] which is similar to 0.81, the value we found in our TOPC data. Another reported that the test-retest correlation of the UPSIT was 0.9 [27] and was 0.86 in the Sniffin’ [28]. These results are of a similar magnitude with our correlation between true and UPSIT equivalent Sniffin’ 16 of 0.8. Both variability in test-retest performance and inadequate conversion may have contributed to the differences between the true and converted scores, though our results are consistent with the test-retest correlations.

There were a number of limitations to our work. The validation dataset we used was small and does not cover the entire range of scores for the two olfaction tests. Also if we had designed our two incidence cohorts with these conversions in mind it would have been better to randomise patients to receive either the UPSIT or the Sniffin’ test. There are also clear differences between the Tracking and Discovery groups, especially in cognition which is related to olfaction, which could be the reason why the equipercentile method on the UPSIT to Sniffin conversion showed some evidence of bias and made it necessary to use the IRT method. Another consideration is that the UPSIT normative data was derived using a US version. The cohorts that we studied used a newer UK version adapted due to cultural differences as some smells in the US version were unfamiliar in the UK population. Despite this, the UK and US versions are still very similar, sharing 33 items with some changes to distractors.

Our UPSIT to B-SIT conversion had high concordance but some evidence of bias. However this disappeared when only considering the PD cases from the TOPC data. None of our other conversions showed evidence of difference in the delta when stratified by PD or Control. This could be because (a) this conversion is not valid; (b) the conversion is valid and the differential observation between PD cases and controls was a chance finding; or (c) our conversion is only valid for PD patients contradicting our belief that differences in olfaction between PD patients and controls is not related to any particular odour type.

The choice of what olfaction test to use in a study will be determined by several factors (i) time available and burden on participants (ii) cost of administering tests (iii) sample size. Another issue to consider is that shorter tests may be less sensitive (e.g. 40-item UPSIT versus 16 item Sniffin’) thereby reducing the ability to differentiate between groups. However statistical power is also related to sample size and measuring the UPSIT on a large sample would take considerably more time than a quicker test like the B-SIT. In some circumstances one may be happy to trade-off sensitivity against increased sample size. Longer tests are also less likely to be affected by random measurement error and will therefore have greater reliability. The association between reliability and test length is most famously highlighted by the Spearman-Brown prediction formula [29] and has been modelled before in olfaction [27]. In olfactory tests this is emphasised by the fact that the test-retest correlation was 0.9 in the UPSIT and 0.71 in the B-SIT [27].

We created a valid and reliable conversion of UPSIT scores to Sniffin’ scores and from Sniffin’ 12 item to 16 item. Also we have arguably created a valid and reliable conversion from UPSIT to B-SIT scores for PD patients. These conversions will be used to merge olfaction data from the Oxford Discovery and Tracking Parkinson's cohorts to investigate the influence of baseline olfaction and hyposmia in predicting future cognitive and motor decline in these longitudinal cohorts of early PD. We believe that these conversion charts will facilitate more replication of research findings and greater data sharing across many neurological diseases and studies that measure olfaction using these tests.

## Funding

The Oxford Discovery study was funded by the Monument Trust Discovery Award from Parkinson's UK (J-0901 and J-1403) and supported by the National Institute for Health Research (NIHR) (HMRWAJO4) Oxford Biomedical Research Centre based at Oxford University Hospitals NHS Trust and University Of Oxford, and the NIHR Clinical Research Network: Thames Valley and South Midlands.

The Tracking Parkinson's study was funded by Parkinson's UK (J-1101) and supported by the National Institute for Health Research (NIHR) DeNDRoN network, the NIHR Newcastle Biomedical Research Unit based at Newcastle upon Tyne Hospitals NHS Foundation Trust and Newcastle University, and the NIHR funded Biomedical Research Centre in Cambridge. The views expressed are those of the authors and not necessarily those of the NHS, the NIHR or the Department of Health.

## Author roles

ML: Data Analysis, manuscript writing and editing.

MTMH and DG: Study design, data collection, manuscript writing and editing.

FB, CR, EB, DMAS, NM: Data collection, manuscript editing.

KAG, NB, RAB, DJB, TF, and HRM: Study design, data collection and manuscript editing.

NW, NWW: Study design and manuscript editing.

MTM: Data analysis, manuscript editing.

YBS: Study design, data analysis and manuscript writing and editing.

## Conflicts of interest

MA Lawton, M Hu, F Baig, C Ruffmann, E Barron, DMA Swallow, N Malek, KA Grosset, N Williams, N Wood, M May, Y Ben-Shlomo: No conflicts of interest.

N Bajaj has received payment for advisory board attendance from UCB, Teva Lundbeck, Britannia, GSK, Boehringer, and honoraria from UCB Pharma, GE Healthcare, Lily Pharma, Medtronic. He has received research grant support from GE Healthcare, Wellcome Trust, MRC and Parkinson's UK and royalties from Wiley.

RA Barker has received grants from Parkinson's UK, NIHR, Cure Parkinson's Trust, Evelyn Trust, Rosetrees Trust, MRC and EU along with payment for advisory board attendance from Oxford Biomedica and LCT, and honoraria from Wiley and Springer.

DJ Burn has received grants from NIHR, Wellcome Trust, GlaxoSmithKline Ltd, Parkinson's UK, and Michael J Fox Foundation. He has acted as consultant for GSK.

T Foltynie has received payment for advisory board meetings for Abbvie and Oxford Biomedica, and honoraria for presentations at meetings sponsored by Medtronic, St Jude Medical, Britannia and Teva pharmaceuticals.

H Morris reports grants from Parkinson's UK, grants from Medical Research Council UK, during the conduct of the study; grants from Welsh Assembly Government, personal fees from Teva, personal fees from Abbvie, personal fees from Teva, personal fees from UCB, personal fees from Boerhinger-Ingelheim, personal fees from GSK, non-financial support from Teva, grants from Ipsen Fund, non-financial support from Medtronic, grants from MNDA, grants from PSP Association, grants from CBD Solutions, grants from Drake Foundation, personal fees from Acorda, outside the submitted work; In addition, H Morris has a patent H. R. M is a co-applicant on a patent application related to C9ORF72 - Method for diagnosing a neurodegenerative disease (PCT/GB2012/052140) pending.

DG Grosset has received payment for advisory board attendance from AbbVie, and honoraria from UCB Pharma, GE Healthcare, and Acorda.

## Figures and Tables

**Table 1:** Demographic and clinical data for Tracking Parkinson's and Discovery cohorts (restricted to recently diagnosed and probability of PD ≥ 90% at latest visit).

Variable	Tracking Parkinson's UPSIT data (N = 980): Mean (sd; range) or n(%)	Tracking Parkinson's Sniffin’ data (N = 294): Mean (sd; range) or n(%)	P-value difference between two tracking Parkinson's groups^e^	Discovery Sniffin’ data (N = 837) mean (sd; range) or n(%)	P-value difference between UPSIT and discovery group	P-value difference between two Sniffin’ groups
Disease duration from diagnosis, years	1.38 (0.9; 0–3.5)	1.14 (0.9; 0–3.1)	<0.001^a^	1.28 (1.0, 0–3.5)	0.02^a^	0.02^a^
Female	347 (35.4%)	101 (34.4%)	0.76^c^	299 (35.7%)	0.89^c^	0.70^c^
Age at test	67.5 (9.1; 31.8–91.1)	67.6 (9.0; 38.1–88.3)	0.93^a^	67.3 (9.5; 32.2–90.5)	0.58^a^	0.62^a^
UPDRS 3	22.1 (11.6; 1–63)	22.1 (12.4; 1–74)	0.84^b^	26.4 (10.9; 5–77)	<0.001^b^	<0.001^b^
Hoehn and Yahr^∗^			0.86^c^		<0.001^c^^,^^d^	<0.001^c^^,^^d^
0–1	508 (52.5%)	143 (49.8%)		193 (23.1%)		
2	417 (43.1%)	132 (46.0%)		581(69.4%)		
3+	43 (4.4%)	12 (4.2%)		63 (7.5%)		
MoCA adjusted	25.4 (3.3; 10–30)	25.4 (3.2; 10–30)	0.93^b^	25.0 (3.3; 13–30)	0.02^b^	0.07^b^
UPSIT score	19.6 (6.7; 3–37)	NA	NA	NA	NA	
Sniffin’ 16 score	NA	7.5 (2.8; 0–15)	NA	7.2 (2.9; 1–15)	NA	0.12^a^
BSIT score	5.7 (2.2; 0–12)	NA	NA	NA	NA	
Sniffin’ 12 score	NA	6.0 (2.4; 0–12)	NA	5.7 (2.5; 0–12)	NA	0.18^a^

UPDRS = Movement Disorder Society unified Parkinson's disease rating scale, MoCA = Montreal cognitive assessment, UPSIT = University of Pennsylvania Smell Identification Test, BSIT = Brief Smell Identification Test.

a T-test.

b Rank-sum test.

c Chi-squared test.

d In Tracking Parkinson's 1.5 changed to 1 and 2.5 changed to 2 for comparability between cohorts.

e One individual with both UPSIT and Sniffin’ in Tracking Parkinson's was excluded from the test of differences between the two groups.

**Table 2:** Conversion table for different methods between the raw UPSIT scores and the equivalent Sniffin’ 16 score.

Raw UPSIT score		Equivalent Sniffin’ 16 score
Equipercentile method	IRT method	
0–3	0–4	0
4–6	5–6	1
7–8	7–8	2
9–10	9–10	3
11–13	11–12	4
14–15	13–14	5
16–17	15–16	6
18–20	17–18	7
21–22	19–21	8
23–24	22–23	9
25–27	24–25	10
28–29	26–27	11
30–32	28–30	12
33–34	31–32	13
35–36	33–35	14
37–38	36–37	15
39–40	38–40	16

**Table 3:** Validation of the different conversions in the Testing of olfaction in Parkinson's and controls (TOPC) validation dataset.

Analysis	Concordance between true score and converted equivalent score	Difference between true score and converted equivalent score mean (sd; range)	Difference between true score and converted equivalent score median (IQR)
Equipercentile method – converting UPSIT to Sniffin’ 16	0.79	0.66 (2.38; −7 to 7)	1 (−1 to 2)
IRT method – converting UPSIT to Sniffin’ 16	0.80	0.14 (2.42; −7 to 7)	0 (−1 to 2)
Equipercentile method – converting Sniffin’ 12 to Sniffin’ 16	0.97	0.01 (0.96; −2 to 2)	0 (−1 to 1)
Equipercentile method – converting UPSIT to BSIT	0.82	−0.63 (1.44; −4 to 2)	−1 (−2 to 0)

**Table 4:** Age and gender stratified 15th centile from UPSIT normative data included smoothed results and the equivalent Sniffin’ results.

Age group	Males			Females		
	≤15th centile UPSIT	Smoothed^a^ ≤15th centile UPSIT	Equivalent Sniffin’	≤15th centile UPSIT	Smoothed^a^ ≤ 15th centile UPSIT	Equivalent Sniffin’
15–19	33	33	14	35	35	14
20–24	33	33	14	35	34	14
25–29	34	33	14	34	34	14
30–34	33	32	13	34	34	14
35–39	33	32	13	34	33	14
40–44	32	31	13	34	33	14
45–49	33	30	12	34	32	13
50–54	29	29	12	32	31	13
55–59	26	27	11	32	30	12
60–64	28	24	10	31	27	11
65–69	22	22	9	26	25	10
70–74	19	19	8	22	22	9
75–79	18	16	6	16	18	7
80–84	12	13	5	15	15	6
>=85	10	9	3	15	13	5

NB. For males 60–64 where the 15th centile is both a score of 28 and 29 we chose 28 which was more in keeping with the surrounding values.

a Smoothed using lowess techniques and a bandwidth of 0.7.
