# Supplementary material for: Equating scores of the University of Pennsylvania Smell Identification Test and Sniffin' Sticks test in patients with Parkinson's disease
Source: Parkinsonism Relat Disord. 2016 Dec;33:96–101. doi: 10.1016/j.parkreldis.2016.09.023 (PMC5159993; doi:10.1016/j.parkreldis.2016.09.023)
Supplement: Supplementary file 1 [file mmc1.docx]

**Equating University of Pennsylvania Smell Identification Test scores and Sniffin’ Scores in Patients with Parkinson’s Disease**

**Supplemental web appendix**

**Methods**

***Statistical Analysis***

To carry out the Item Response Theory method (IRT) we fitted a series of latent variable models for each item where olfaction is the latent variable. We modelled the probability of correctly answering an item on a test given the latent olfaction variable and a three parameter IRT model. These parameters are often described as the discrimination, difficulty and lower asymptote parameters. An example of an IRT model can be seen in web figure 1. Here *θ* is the latent olfaction variable; *c* is the lower asymptote parameter, or equivalently the probability of correctly answering the question even with very poor olfaction or by chance; *b* is the difficulty parameter or level of olfaction at which the probability is exactly half-way between 1 and the lower asymptote; *a* is the discrimination parameter or proportional to the slope where *θ* = *b*. Within a group we scale the olfaction variable to have a mean of zero and standard deviation of 1.

After creating the IRT model we calibrated the two sets of models using the fact that the estimated parameters should be identical in the common items if the olfaction of the two groups is also identical. In this study we assumed that items were common if they have the same answer. For instance the correct answer for item 11 on the Sniffin’ and item 20 on the UPSIT is apple. Using this definition there are 13 common items between the Sniffin’ and UPSIT. There are a number of methods that can be used to calibrate the items on two tests, in our case we used the Stocking-Lord characteristic curve method. Once we have fitted our models and calibrated the parameters between the two tests, then we can equate the overall scores on the two tests using either the true or observed score equating methods, in our case we used the observed score equating method. We decided to use the observed score method because theoretically its properties are easier to justify and it does not require extrapolation at the very low scores where the true score method is undefined.

The equipercentile equating was carried out using the equate library [1] in R 3.0.1. The IRT model fitting was carried out in BILOG-MG [2], the calibration using STUIRT [3] and the observed score equating using PIE [4].

**Discussion**

We used the observed score equating method as part of our IRT equating. However the other IRT equating method, true score equating, would have given slightly different conversions especially at the upper and lower ends of the scales. However when validating the true score equating on the TOPC data we found very similar results with a small bias between true and converted Sniffin’ results (results not shown - available on request). Also of note is that as well as combining the Sniffin’ data for the UPSIT to Sniffin’ conversion we also tried each method using the Sniffin’ data from each cohort separately (results not included). This led to nearly identical results and for brevity we only included the combined analysis.

There are some limitations to this work in our application of the IRT method. Firstly there was evidence of a lack of fit in some questions but BILOG warns that the item fit statistic might be unreliable when the number of items is less than 20. This is exactly what we observed with a greater lack of fit in the Sniffin’ test compared to the UPSIT. Secondly we had some slight convergence issues for the Sniffin’ test which is likely to be due to the smaller number of items. Thirdly we made the tenuous assumption that the items on the Sniffin’ and UPSIT tests are “common items” if the answers are the same. How a question functions in a test (which would affect estimates of the three IRT parameters) is likely to be related to not only the correct answer but also the three distractors which were not always the same across the two tests. Fourthly the different calibration methods in IRT gave different estimates for the calibration slope and intercept which is likely due to the problems around the “common items” assumption. However despite these limitations it is reassuring that the validation of our conversion showed good characteristics.

On a practical perspective, the equipercentile method was very simple to carry out within R. In contrast the time taken to learn and carry out IRT equating was considerably longer given the need to use multiple programs and the difficulty of exporting data in different formats between these programs.

**Web Figures legends**

Web Figure 1. Example of a three parameter logistic IRT model. The dashed lines are added to help conceptualise the three parameters.

Web Figure 2. Distribution of Sniffin’ 16 and UPSIT scores in the Testing of olfaction in Parkinson’s and controls (TOPC) validation dataset, stratified by patient type.

Web Figure 3. Agreement of true Sniffin’ 16 and UPSIT equivalent Sniffin’ 16 in the Testing of olfaction in Parkinson’s and controls validation dataset using the two conversion methods. Size of dots are proportional to the number of individuals with that score.

Web Figure 4. Agreement of true Sniffin’ 16 and Sniffin’ 12 equivalent Sniffin’ 16 in the Testing of olfaction in Parkinson’s and controls validation dataset. Size of dots are proportional to the number of individuals with that score.

Web Figure 5. Agreement of true B-SIT and UPSIT equivalent B-SIT in the Testing of olfaction in Parkinson’s and controls validation dataset. Size of dots are proportional to the number of individuals with that score.

**References web appendix**

[1] Albano A. Equate: Observed-Score Linking and Equating. (Version 2.0-3 R package) 2014. Available from http://CRAN.R-project.org/package=equate.

[2] Scientific Software International. BILOG-MG for Windows. (Version 3.0.2327.2) 2003. Available from http://www.ssicentral.com/.

[3] Kim S, Kolen MJ. STUIRT - A computer program for Scale Transformation under Unidimensional Item Response Theory Models. (Version 1.0) 2004. Available from http://education.uiowa.edu/centers/center-advanced-studies-measurement-and-assessment/computer-programs

[4] Hanson B, Zeng L, Cui Z. PIE - A computer program for IRT Equating. (Windows Console version) 2004. Available from http://education.uiowa.edu/centers/center-advanced-studies-measurement-and-assessment/computer-programs
