# Supplementary material for: Equating scores of the University of Pennsylvania Smell Identification Test and Sniffin' Sticks test in patients with Parkinson's disease
Source: Parkinsonism Relat Disord. 2016 Dec;33:96–101. doi: 10.1016/j.parkreldis.2016.09.023 (PMC5159993; doi:10.1016/j.parkreldis.2016.09.023)
Supplement: Supplementary file 2 [file mmc2.docx]

Web table 1. Demographic and olfaction data for the Testing of olfaction in Parkinson’s and controls (TOPC) validation dataset

| **Variable** | **Controls (N=67)**  **mean (sd; range) or n (%)** | **PD (N=61)**  **mean (sd; range) or n (%)** |
| --- | --- | --- |
| **Female** | 36 (53.7%) | 24 (39.3%) |
| **Age at test** | 61.0 (13.2; 18.0 – 81.8) | 66.8 (8.9; 46.5-81.6) |
| **UPSIT** | 28.0 (6.9; 6-39) | 16.7 (6.0; 7 – 38) |
| **Sniffin’ 16** | 11.6 (2.8; 5-16) | 6.6 (3.3; 2-15) |
| **BSIT** | 7.3 (2.2; 3- 11) | 4.7 (2.1; 1-11) |
| **Sniffin’ 12** | 9.3 (2.4; 4-12) | 5.3 (2.6; 2-11) |
| **Order (took UPSIT first)** | 34 (50.8%) | 33 (54.1%) |
