# Supplementary material for: Equating scores of the University of Pennsylvania Smell Identification Test and Sniffin' Sticks test in patients with Parkinson's disease
Source: Parkinsonism Relat Disord. 2016 Dec;33:96–101. doi: 10.1016/j.parkreldis.2016.09.023 (PMC5159993; doi:10.1016/j.parkreldis.2016.09.023)
Supplement: Supplementary file 3 [file mmc3.docx]

Web table 2. IRT parameter estimates for three-parameter logistic IRT model using UPSIT data of 980 individuals. The chi-square item fit p-value is a test between observed and predicted proportions so a small p-value represents lack of fit.

| **Item** | **Proportion correct** | **a**  **(discrimination)** | **b**  **(difficulty)** | **c**  **(lower asymptote)** | **Chi-square item fit**  **p-value** |
| --- | --- | --- | --- | --- | --- |
| 1 | 0.249 | 1.004 | 1.795 | 0.161 | 0.966 |
| 2 | 0.440 | 0.648 | 1.610 | 0.305 | 0.627 |
| 3 | 0.589 | 1.004 | 0.063 | 0.205 | 0.186 |
| 4 | 0.408 | 0.767 | 1.191 | 0.226 | 0.959 |
| 5 | 0.438 | 0.634 | 0.781 | 0.154 | 0.484 |
| 6 (mint) | 0.543 | 1.113 | 0.394 | 0.253 | 0.528 |
| 7 (banana) | 0.496 | 1.087 | 1.290 | 0.384 | 0.981 |
| 8 (clove) | 0.522 | 1.291 | 0.683 | 0.316 | 0.901 |
| 9 (leather) | 0.681 | 0.851 | -0.291 | 0.246 | 0.217 |
| 10 | 0.442 | 1.169 | 1.075 | 0.290 | 0.967 |
| 11 | 0.758 | 0.835 | -0.829 | 0.188 | 0.036 |
| 12 | 0.210 | 1.296 | 1.616 | 0.119 | 0.614 |
| 13 | 0.792 | 1.040 | -0.895 | 0.203 | 0.008 |
| 14 (coffee) | 0.558 | 0.500 | 0.278 | 0.198 | 0.689 |
| 15 (cinnamon) | 0.430 | 1.146 | 1.534 | 0.342 | 0.694 |
| 16 | 0.383 | 0.906 | 1.007 | 0.176 | 0.672 |
| 17 | 0.545 | 0.918 | 0.248 | 0.196 | 0.599 |
| 18 | 0.476 | 0.945 | 0.965 | 0.293 | 0.571 |
| 19 | 0.632 | 0.757 | -0.263 | 0.158 | 0.204 |
| 20 (apple) | 0.533 | 0.790 | 1.115 | 0.376 | 0.993 |
| 21 | 0.664 | 0.580 | -0.376 | 0.207 | 0.234 |
| 22 (turpentine) | 0.233 | 0.935 | 2.447 | 0.191 | 0.655 |
| 23 | 0.612 | 0.811 | -0.061 | 0.200 | 0.091 |
| 24 (liquorice) | 0.331 | 1.404 | 1.214 | 0.197 | 0.831 |
| 25 | 0.195 | 0.861 | 3.124 | 0.175 | 0.961 |
| 26 (pineapple) | 0.495 | 0.738 | 0.763 | 0.250 | 0.49 |
| 27 | 0.333 | 0.623 | 2.492 | 0.260 | 0.956 |
| 28 (orange) | 0.532 | 0.859 | 0.502 | 0.252 | 0.478 |
| 29 | 0.519 | 0.816 | 0.627 | 0.263 | 0.992 |
| 30 | 0.445 | 0.880 | 0.888 | 0.228 | 0.652 |
| 31 | 0.388 | 0.611 | 1.470 | 0.214 | 0.71 |
| 32 | 0.382 | 0.981 | 1.563 | 0.279 | 0.764 |
| 33 | 0.730 | 1.135 | -0.447 | 0.266 | 0.07 |
| 34 | 0.559 | 1.115 | 0.675 | 0.356 | 0.998 |
| 35 | 0.750 | 1.118 | -0.637 | 0.216 | 0.033 |
| 36 (lemon) | 0.291 | 0.803 | 2.477 | 0.241 | 0.218 |
| 37 | 0.403 | 0.487 | 3.013 | 0.337 | 0.953 |
| 38 | 0.536 | 0.956 | 0.250 | 0.183 | 0.222 |
| 39 (rose) | 0.460 | 0.850 | 0.790 | 0.222 | 0.691 |
| 40 | 0.608 | 1.041 | -0.043 | 0.196 | 0.263 |
