# Supplementary material for: Equating scores of the University of Pennsylvania Smell Identification Test and Sniffin' Sticks test in patients with Parkinson's disease
Source: Parkinsonism Relat Disord. 2016 Dec;33:96–101. doi: 10.1016/j.parkreldis.2016.09.023 (PMC5159993; doi:10.1016/j.parkreldis.2016.09.023)
Supplement: Supplementary file 4 [file mmc4.docx]

Web Table 3. IRT parameter estimates for three-parameter IRT model using combined visit 1 Sniffin’ data. The chi-square item fit p-value is a test between observed and predicted proportions so a small p-value represents lack of fit.

| **Item** | **Proportion correct** | **a**  **(discrimination)** | **b**  **(difficulty)** | **c**  **(lower asymptote)** | **Chi-square item fit**  **p-value** |
| --- | --- | --- | --- | --- | --- |
| 1 (orange) | 0.714 | 0.747 | -0.525 | 0.186 | <0.001 |
| 2 (leather) | 0.452 | 0.823 | 1.229 | 0.320 | 0.269 |
| 3 (cinnamon) | 0.350 | 0.596 | 1.868 | 0.262 | 0.317 |
| 4 (mint) | 0.677 | 0.790 | -0.176 | 0.236 | <0.001 |
| 5 (banana) | 0.446 | 0.795 | 0.754 | 0.208 | 0.003 |
| 6 (lemon) | 0.354 | 0.664 | 1.781 | 0.175 | 0.08 |
| 7 (liquorice) | 0.469 | 1.666 | 0.924 | 0.218 | 0.04 |
| 8 (turpentine) | 0.347 | 0.557 | 3.823 | 0.315 | 0.834 |
| 9 | 0.585 | 0.584 | 0.526 | 0.321 | 0.01 |
| 10 (coffee) | 0.490 | 0.867 | 0.968 | 0.186 | 0.07 |
| 11 (apple) | 0.190 | 1.098 | 2.861 | 0.154 | 0.427 |
| 12 (clove) | 0.442 | 0.662 | 0.870 | 0.231 | 0.043 |
| 13 (pineapple) | 0.364 | 0.562 | 1.283 | 0.171 | 0.031 |
| 14 (rose) | 0.561 | 0.375 | 0.804 | 0.252 | 0.055 |
| 15 | 0.381 | 2.257 | 0.970 | 0.185 | 0.213 |
| 16 | 0.639 | 1.112 | -0.166 | 0.195 | <0.001 |
