# Supplementary material for: Equating scores of the University of Pennsylvania Smell Identification Test and Sniffin' Sticks test in patients with Parkinson's disease
Source: Parkinsonism Relat Disord. 2016 Dec;33:96–101. doi: 10.1016/j.parkreldis.2016.09.023 (PMC5159993; doi:10.1016/j.parkreldis.2016.09.023)
Supplement: Supplementary file 5 [file mmc5.docx]

Web Table 4. Conversion from Sniffin’ 12 score to Sniffin’ 16.

| **Raw Sniffin’ 12 score** | **Equivalent Sniffin’ 16 score** |
| --- | --- |
| 0 | 1 |
| 1 | 2 |
| 2 | 3 |
| 3 | 4 |
| 4 | 5 |
| 5 | 6 |
| 6 | 7 |
| 7 | 9 |
| 8 | 10 |
| 9 | 11 |
| 10 | 12 |
| 11 | 14 |
| 12 | 15 |
