# Supplementary material for: Equating scores of the University of Pennsylvania Smell Identification Test and Sniffin' Sticks test in patients with Parkinson's disease
Source: Parkinsonism Relat Disord. 2016 Dec;33:96–101. doi: 10.1016/j.parkreldis.2016.09.023 (PMC5159993; doi:10.1016/j.parkreldis.2016.09.023)
Supplement: Supplementary file 6 [file mmc6.docx]

Web Table 5. Conversion from UPSIT score to B-SIT scores

| **Raw UPSIT score** | **Equivalent B-SIT score** |
| --- | --- |
| 0 - 4 | 0 |
| 5 - 7 | 1 |
| 8 - 10 | 2 |
| 11 - 12 | 3 |
| 13 - 15 | 4 |
| 16 - 18 | 5 |
| 19 - 21 | 6 |
| 22 - 24 | 7 |
| 25 - 28 | 8 |
| 29 - 31 | 9 |
| 32 - 35 | 10 |
| 36 - 38 | 11 |
| 39 - 40 | 12 |
