# Supplementary material for: Equating scores of the University of Pennsylvania Smell Identification Test and Sniffin' Sticks test in patients with Parkinson's disease
Source: Parkinsonism Relat Disord. 2016 Dec;33:96–101. doi: 10.1016/j.parkreldis.2016.09.023 (PMC5159993; doi:10.1016/j.parkreldis.2016.09.023)
Supplement: Supplementary file 8 [file mmc10.docx]

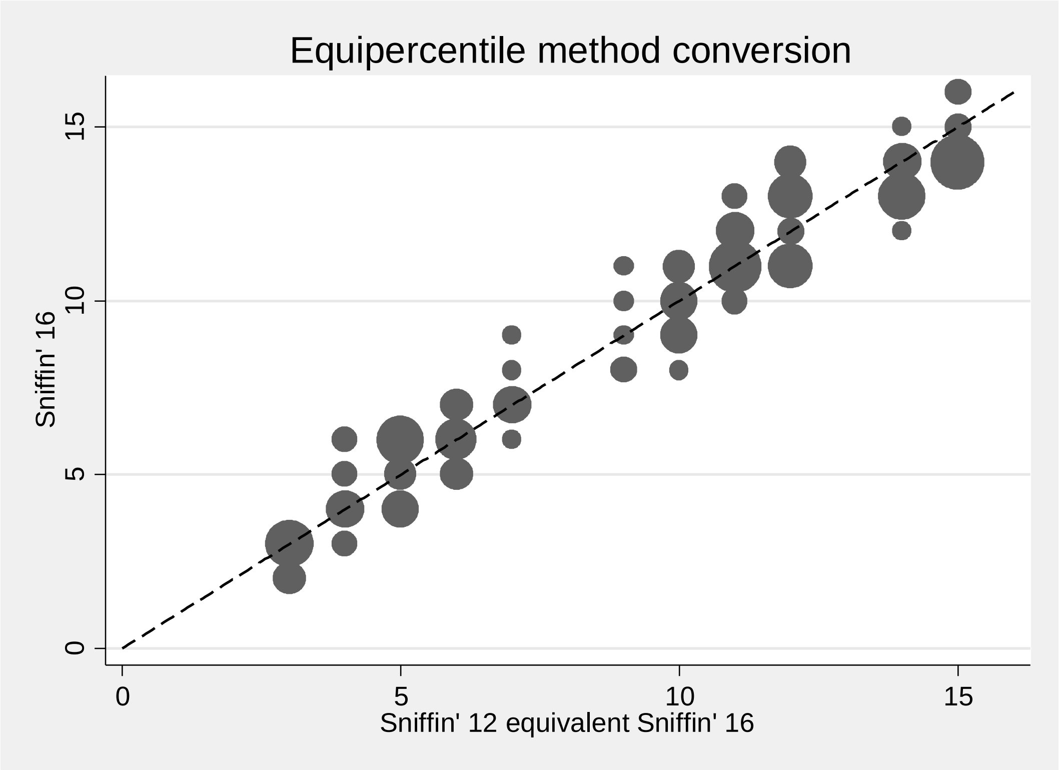
Equipercentile method conversion
Sniffin' 16
0
5
10
15
0
5
10
15
Sniffin' 12 equivalent Sniffin' 16
